# Exploring cholinergic dysfunctions in schizophrenia as targets for a new generation of antipsychotics- The current level of evidence

**DOI:** 10.1192/j.eurpsy.2025.2104

**Published:** 2025-08-26

**Authors:** O. Vasiliu, A. M. Ciobanu, A. G. Mangalagiu, B. M. Petrescu

**Affiliations:** 1 Neuroscience Department, “Carol Davila” University of Medicine; 2 Psychiatry Department, “Dr. Carol Davila” University Emergency Central Military Hospital; 3 Psychiatry Department, “Prof. Dr. Alexandru Obregia” Clinical Hospital of Psychiatry, Bucharest, Romania

## Abstract

**Introduction:**

The dopaminergic hypothesis of schizophrenia has been constantly revised and virtually all the antipsychotics currently available for the treatment of schizophrenia are based on this pathogenetic hypothesis. However, the involvement of cholinergic, glutamate, gamma-amino-butyric acid, serotonin, neurotrophins, and pro-inflammatory cytokines in the onset of psychotic disorders is attracting more and more interest. This interest is fueled by the high rates of treatment resistance in schizophrenia, which reaches 15-40% (Wong *et al* Transl Psychiatry 2024;50 14) and requires second-line treatment, frequently associated with significant adverse events. Finding new pharmacological agents that can be used either as monotherapy or as add-ons to the ongoing treatment in patients with schizophrenia is essential for improving the chances of functional recovery.

**Objectives:**

To review the evidence supporting the modulation of cholinergic neurotransmission as a potential pharmacological target for treating schizophrenia.

**Methods:**

Two clinical studies repositories (US National Library of Medicine- clinicaltrials.gov and WHO International Clinical Trials Registry Platform-https://www.who.int/clinical-trials-registry-platform) and the PubMed database were explored using “choline*” and “schizophrenia” or “psychotic disorders” or “schizophrenia spectrum disorders” for studies and reviews focused on cholinergic agents targeting symptoms of schizophrenia.

**Results:**

Positive allosteric modulation of the α7 nicotine receptors and M1/M4 muscarinic receptor agonism are the two pharmacodynamic mechanisms explored for cholinergic-based antipsychotics. Other possible mechanisms of interest are positive allosteric modulation of the M5 muscarinic receptors, selective M4 positive allosteric modulation, and cholinesterase inhibition. Out of these preclinically explored options, only xanomeline, an M1/M4 muscarinic receptor agonist, has reached phase III of clinical research with significant antipsychotic effects. The currently explored formula is an association of a fixed dose of xanomeline (selective M1/M4 cholinergic agonist) and trospium (a cholinergic antagonist), the last agent being included to decrease the risks of peripheric cholinergic adverse events. Studies investigating cholinesterase inhibitors have not been associated with favorable results, and the tolerability was low. Positive allosteric modulators of the α7 nicotine receptors are investigated in preclinical studies, but in phase 1b such an agent, i.e., AVL-3288, failed to show efficacy versus placebo.

**Conclusions:**

The association of xanomeline, an M1/M4 receptor agonist, and trospium, a peripheric cholinergic antagonist, led to favorable results in phase III trials. Other molecules with cholinergic mechanisms are also explored in schizophrenia, but the results are not yet clinically significant.

**Disclosure of Interest:**

None Declared

